# Erratic Male Meiosis Resulting in 2*n* Pollen Grain Formation in a 4x Cytotype (2*n* = 28) of *Ranunculus laetus* Wall. ex Royle

**DOI:** 10.1100/2012/691545

**Published:** 2012-05-02

**Authors:** Puneet Kumar, Vijay Kumar Singhal

**Affiliations:** Department of Botany, Punjabi University, Patiala 147 002, Punjab, India

## Abstract

Two accessions were studied for male meiosis in *Ranunculus laetus* from the cold regions of Northwest Himalayas. One accession showed the presence of 14 bivalents at diakinesis and regular segregation of bivalents at anaphase I which lead to normal tetrad formation with four *n* microspores and consequently *n* pollen grains and 100% pollen fertility. Second accession from the same locality revealed the erratic meiosis characterized by the presence of all the 28 chromosomes as univalents in meiocytes at metaphase I. Univalent chromosomes failed to segregate during anaphases and produced restitution nuclei at meiosis I and II. These restitution nuclei resulted into dyads and triads which subsequently produced two types of apparently fertile pollen grains. On the basis of size, the two types of pollen grains were categorized as *n* (normal reduced) and 2*n* (unreduced, 1.5-times larger than the *n* pollen grains). The estimated frequency of 2*n* pollen grains from dyads and triads (61.59%) was almost the same as that of the observed one (59.90%), which indicated that 2*n* pollen grains in *R. laetus* were the result of dyads and triads. The present paper herein may provide an insight into the mechanisms of the formation of various intraspecific polyploids through sexual polyploidization in *R. laetus*.

## 1. Introduction

*Ranunculus laetus* Wall. ex Royle (family: Ranunculaceae), a highly polymorphic species [1, 2], has been studied chromosomally quite extensively from various regions of the Himalayas in India and outside of India from the hills of Nepal, Russia, China, and Pakistan (Figures 1(a) and 1(b)). The species exhibited a great amount of heterogeneity in chromosome number and level of ploidy with 2x ((2*n* = 14) [3], Cangshan Mountains, Yunnan, China; (2*n* = 16) [4], Nepal), 4x ((2*n* = 28), [1, 5, 6], Kashmir Himalayas; [7, 8], Eastern Himalayas; [9], Northwest Himalayas; [10, 11], Russia; [12, 13], Garhwal Himalayas; [14], Kinnaur in Himachal Pradesh; [15, 16], Chamba, Lahaul-Spiti, Kinnaur and Dalhousie hills in Himachal Pradesh; (2*n* = 32) [1], Shimla hills in Himachal Pradesh; [17], Indian Himalayas; [18], Kashmir Himalayas; [19, 20], Western Pakistan; [21, 22] Pakistan), 6x ((2*n* = 42) [8], Eastern Himalayas), 8x ((2*n* = 56) [20], Pakistan) based on two different basic chromosome numbers (2x, 4x, 6x, 8x on x = 7, 2x, 4x on x = 8).

Despite these intraspecific chromosomal variations and levels of ploidy (2x, 4x, 6x, and 8x) nothing is known about the origin of various polyploids in the species. All the previous studies carried out in the species were restricted either to count the chromosome number or to study the karyotype or DNA content. Previous communications [15, 16] from this laboratory have addressed in detail the cytological behaviour in 12 different accessions from the cold deserts of India, focusing on male meiosis. These accessions which uniformly existed at 4x level (2*n* = 28) showed normal bivalent formation and equal segregation of chromosomes at anaphases. However, these accessions depicted some irregularities during male meiosis such as cytomixis, chromosome stickiness, pycnotic chromatin material, out of plate bivalents at metaphase I, nonsynchronous disjunction of bivalents, and laggards at anaphases/telophases which resulted into PMCs (pollen mother cells) with abnormal microsporogenesis and 9–31% pollen sterility [16]. While studying the male meiosis in the species from the cold regions of Northwest Himalayas in Chamba district we have noticed in one accession that all the 28 chromosomes remained as univalents in the PMCs at metaphase I (M-I) during meiosis I. The products of such PMCs produced restitution nuclei and consequently yielded 2*n* (unreduced) pollen grains.

The present study herein aims to analyze the detailed meiotic course, microsporogenesis and to elucidate the cytological mechanism that lead to the formation of 2*n* pollen grains. The study may also provide an insight into the mechanisms of the formation of various intraspecific polyploids in *R. laetus*.

## 2. Material and Methods

### 2.1. Plant Material

Material for male meiotic studies was collected from the wild plants growing on open moist slopes around the apple orchards in Bharmour in Chamba district (32°26′24′′N, 76°33′31, altitude, 2,350 m) of Himachal Pradesh in June-July of 2009. The cytologically worked-out plants were identified using regional floras and compared with the specimens deposited at the Herbarium of Botanical Survey of India, Northern Circle, Dehra Dun. The voucher specimens (PUN, 51345, 51346) were deposited in the Herbarium, Department of Botany, Punjabi University, Patiala (PUN).

### 2.2. Meiotic Studies

For meiotic chromosome counts, unopened floral buds of suitable sizes were fixed in a freshly prepared Carnoy's fixative (mixture of alcohol, chloroform, and glacial acetic acid in a volume ratio 6 : 3 : 1) for 24 h. These were subsequently transferred to 70% alcohol and stored in refrigerator at 4°C until used for meiotic analysis. Meiocytes were prepared by squashing the developing anthers, and stained with acetocarmine (1%). Chromosome number was determined at M-I from freshly prepared slides with light microscope Olympus. 500−600 pollen mother cells were analyzed for meiotic behaviour at different stages, M-I/II, anaphase I/II (A-I/II), telophase I/II (T-I/II).

### 2.3. Pollen Grain Analysis

Pollen fertility was estimated through stainability tests using glycerol-acetocarmine (1 : 1) mixture and aniline blue (1%). Up to 450−800 pollen grains were examined for pollen fertility and size frequencies. Well-filled pollen grains with stained nuclei were taken as apparently fertile while shriveled and unstained pollen were counted as sterile. In each case, the size of 200 pollen grains was measured using an occulomicrometre. As per Xue et al. [23] pollen grain which measures 1.5-times larger than the *n* (normal reduced) pollen in diameter was taken as 2*n* (unreduced) pollen. Estimation of the theoretical frequency of 2*n* pollen grains was made from the number of observed dyads, triads, and tetrads during microsporogenesis. Generally a dyad resulted into two unreduced pollen grains, a triad produced one unreduced pollen grain and two reduced pollen grains, and each tetrad gave rise to four reduced pollen grains. The frequency of 2*n* pollen grains was calculated following Xue et al. [23]:

(1) $$\begin{array}{cc} \text{Frequency}\;\text{of}\;2n\;\text{pollen}\;\text{grains} & =\frac{2\times \text{dy}+\text{tri}}{2\times \text{dy}+3\times \text{tri}+4\times \text{tet}},\\ & \;\times 100\% \end{array}\;$$

dy = total number of dyads observed; tri = total number of triads observed; tet = total number of tetrads observed.

### 2.4. Photomicrographs

Photomicrographs from the freshly prepared desirable slides having clear chromosome counts, dyads, triads, tetrads, and pollen grains were taken with a digital imaging system of *Leica QWin.*

## 3. Results

Meiosis in one of the accession collected from Bharmour, 2,300 m was totally normal with the presence of 14 bivalents at diakinesis (Figure 2(a)) and regular 14 : 14 segregation of chromosomes at opposite poles (Figures 2(a) and 2(c)) leading to normal tetrads with four *n* microspores (Figure 2(d)) and consequently *n* pollen grains (21.98–26.35 *μ*m × 20.82–24.01 *μ*m, Figure 2(e)) and 100% pollen fertility. However, the second accession also collected from Bharmour, 2,300 m showed highly abnormal meiosis characterized by the erratic behaviour of chromosomes at different stages of meiosis I and II.

### 3.1. Chromosomal Behaviour during Meiosis I

Analysis of PMCs at M-I of meiosis I revealed that all the PMCs showed the presence of 28 chromosomes as univalents which either remained randomly dispersed in the cytoplasm or shifted towards the periphery or in the centre or in 2–5 groups in the PMCs (Figures 3(a)–3(c)). Furthermore, the movements of chromosomes at A-I is very irregular, and in most of the PMCs they lagged behind (57.83%, Figure 3(d)). In the majority of the cases these lagging chromosomes did not get included into the telophase nuclei and formed micronuclei at T-I (Figure 3(e)). In some of the PMCs, segregation of chromosomes at A-I was irregular and the most common distribution was observed to be 11 : 17 (Figure 3(f)). In many PMCs it was also noticed that chromosome failed to move towards the A-I poles and remained in the centre of the PMC to form restitution nuclei (Figures 3(g) and 3(h)). Even some of the PMCs showed thick chromatin bridges at anaphases and telophases which did not allow the separation of chromatin material and thus formed restitution nuclei (Figure 3(i)).

### 3.2. Chromosomal Behaviour during Meiosis II

Behaviour of chromosomes during different stages of meiosis II was also erratic and was characterized by the irregular segregation of chromosome at two poles of M-II. The most common distribution of chromosomes in the PMCs at M-II was 10 : 18 (Figure 4(a)). In some of the PMCs the chromosome remained scattered and unoriented during M-II and anaphase II (Figures 4(b) and 4(c)). Some of the chromosomes (1–6) lagged behind at A-II (62.69%, Figure 4(d)) and did not get included in four haploid nuclei at T-II and constituted micronuclei during sporad stage (Figure 4(e)). Formation of restitution nuclei at second meiotic division can be seen in Figure 4(f) where one PMC showed four haploid nuclei at T-II and the adjacent PMC with only two restitution (unreduced) nuclei which probably resulted into dyad formation as evidenced from the presence of the dyads during microsporogenesis. Analysis of 1445 sporads during microsporogenesis revealed the presence of dyads in 73.77% (1066/1445) of the observed sporads (without micronuclei 299/1445, 20.70%, Figure 4(g), or with micronuclei 767/1445, 53.07%, Figure 4(g)) or, triads (71/1445, 4.91%, Figure 4(g)) and tetrads with micronuclei (308/1445, 21.32%, Figure 4(g)).

### 3.3. 2*n* (Unreduced) Pollen Grain Formation

Although pollen fertility was not affected significantly (92%, Figure 4(h)), the erratic meiotic behaviour resulted in two sizes of pollen grains. Depending on the size, these apparently fertile pollen grains were categorized as *n* (22.48–27.98 *μ*m × 19.27–24.77 *μ*m, normal reduced) and 2*n* (29.82–33.49 *μ*m × 25.23–32.56 *μ*m, unreduced; Figure 4(i)). The 2*n* pollen grains were noticed to be in higher frequency (59.90%) compared to the *n* pollen grains (40.10%).

Each dyad give rise to two 2*n* microspores whereas a triad produced only one 2*n* microspore and two *n* microspores. The frequency of apparently fertile 2*n* pollen grains which was estimated from different types of sporads found to be 61.59%. The frequency of 2*n* pollen grains estimated collectively from dyads and triads was almost near the observed one, 59.90% which indicated that the 2*n* pollen grains in *R. laetus* were resulted from dyads and triads at sporad stage which originated from the restitution nuclei observed during meiosis I and II.

## 4. Discussion

The chromosome number in sexually reproducing eukaryotes does not get doubled at each generation which is ensured through a precise, systematic, and specialized process of meiosis [24]. Vital events of this dynamic process are the recognition of homologues chromosomes and their subsequent pairing and synapsis, which are the prerequisites for genetic recombination and balanced gamete formation. Successful completion of meiosis relies on the above mentioned events during the cell cycle. Interactions between homologous chromosomes during recognition, pairing, and synapsis are highly coordinated and controlled by a large number of genes [25–29]. Dysfunctioning of any one of these events generally resulted in serious consequences like failure of chromosome pairing which may have resulted in unbalanced gamete formation. Synaptic mutants represent one such event in the cell cycle where homologous chromosomes either lack pairing during late prophase I [30] or they are not able to generate or retain chiasmata [26, 31, 32]. To describe the condition where homologous chromosomes failed to pair, the term asynapsis is employed. On the other hand, in cases where chromosomes paired at zygotene and pachytene but failed to remain paired during subsequent stages of meiosis refers to desynapsis. In the present investigation all the analysed PMCs did not reveal the expected chromosome associations of 14II, instead they exhibited completely random dispersion of univalents in the cytoplasm at M-I suggesting asynaptic mutation. Peirson et al. [33] were of the opinion that in most of the asynaptic mutants univalents show random distribution in the cytoplasm at M-I and never align at the equatorial plate while in desynapsis bivalents and univalents orient at the equatorial plate during M-I.

 Synaptic variation resulting in complete and partial failure of chromosome pairing of homo/homeologous chromosomes has been studied in a large number of species [15, 26, 34–38]. Physical and chemical mutagens are widely reported to induce synaptic mutations [39–41] but only a few reports are available on the spontaneous origin of synaptic variants in natural populations [36–38, 42, 43]. A large number of factors such as drastic temperature fluctuation, ageing, water content and humidity, soil conditions, and gene mutations [26, 43–45] are reported to be responsible for the spontaneous origin of synaptic mutants in natural populations. The accession with completely normal meiotic behaviour and 100 percent pollen fertility was growing along with the individual which showed synaptic mutation. So the genetic factors seem plausible behind the synaptic irregularities in the species.

 Another interesting phenomenon in the presently investigated species is the formation of restitution nuclei. Restitution nuclei were formed because univalent chromosomes/daughter chromatids failed to distribute themselves uniformly at the poles during anaphases. These restitution nuclei resulted into the formation of dyads and triads which subsequently produced two types of pollen grains. Different methods had been used to detect production of 2*n* pollen grains in plants. Owing to the relatively close correlation between larger pollen grains and 2*n* status, the presence of large-sized pollen grains had been frequently used as a criteria for the indication of 2*n* pollen [23, 46–52]. Presently, on the basis of size, two types of pollen grains were categorized as *n* (normal reduced) and 2*n* (unreduced). The pollen grains which were 1.5-times larger than the normal pollen were regarded here as 2*n* pollen grains. Similar criteria to distinguish between *n* and 2*n* pollen grains had been used earlier by Peng [53] and Xue et al. [23] while studying the 2*n* pollen formation in Chinese jujube. The exact chromosome number of such double-sized pollen grains could not be ascertained during the present investigations but these were surely of the unreduced in their genetic constitution as is clearly depicted from their size, as increasing nucleus and cytoplasm content may in turn influence pollen diameter [50, 54–58]. The estimated frequency of 2*n* pollen grains from dyads and triads was almost the same as that of the observed one, which indicated that the 2*n* pollen grains in *R. laetus* were the result of dyads and triads at sporad stage which originated from the restitution nuclei formed during meiosis I and II.

 The large-sized 2*n* pollen grains were observed to be well filled, stained, and apparently fertile; therefore, it is very much possible that fertilization by these 2*n* gametes can produce intraspecific polyploids [15, 59–63]. The formation of 2*n* gametes is a common phenomenon in the plants which may result from a variety of different meiotic irregularities [63–65]. Unreduced gametes (2*n* pollen grains) or gametes with somatic chromosome number are considered one of the main processes for natural polyploidization of plants. These 2*n* pollen grains may play an important role in the establishment of new polyploid genotypes as suggested by Dewitte et al. [66] and Silva et al. [67]. Unreduced gametes are of colossal significance in cytogenetics as well as applied plant breeding and facilitate the production of new polyploid species [23]. The main advantage which 2*n* pollen grains offer over asexual polyploidization is the transmission of the parental heterozygosity to the offspring. The 2*n* gametes produced through restitution nuclei can transfer at least 75–80% heterozygosity [47, 68].

 In a number of plants earlier workers have reported that synaptic mutation causes pollen sterility [15, 31, 36–38, 69–72]. However, in the present study pollen fertility was not affected seriously and was quite high (92%) which may be due the fact that dyads produced through restitution nuclei are genetically balanced which lead to a higher degree of pollen fertility. Similar observations regarding the high pollen fertility in an asynaptic mutant of *Allium amplectens *had been made by Levan [73]. The presence of some pollen sterility (8%) could be attributed to the presence of unoriented univalents which lag during anaphases/telophases, and constitute micronuclei at sporad stage.

## 5. Conclusions

Presently studied accession with erratic male meiosis is a spontaneous asynaptic mutant in which univalent chromosomes behaved in a highly irregular manner resulting into restitution nuclei and consequently 2*n* pollen grains. Furthermore, authors safely conclude that 2*n* pollen grains may have played a role in the evolution of species by forming the polyploid genotypes through sexual polyploidization as has been suggested by others [59, 62, 74, 75].

## Figures and Tables

**Figure 1:**
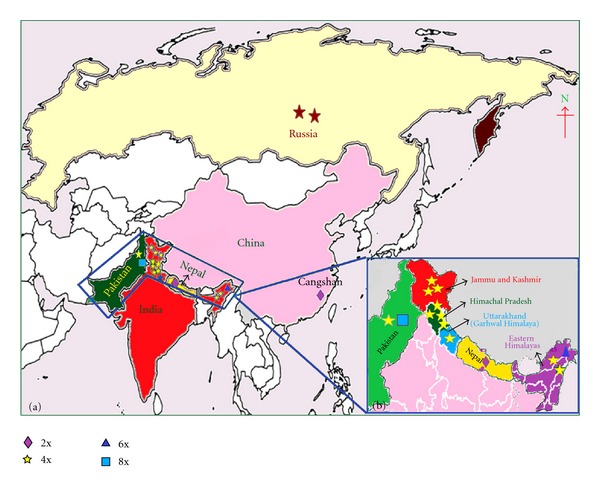
(a) Map showing distribution of 2x, 4x, 6x, and 8x (indicated with symbols) cytotypes in India, China, Nepal, Pakistan, and Russia. (b) Distribution of different cytotypes in Himalayan regions of India (2x, 4x, 6x), Nepal (2x), and Pakistan (4x, 8x).

**Figure 2:**
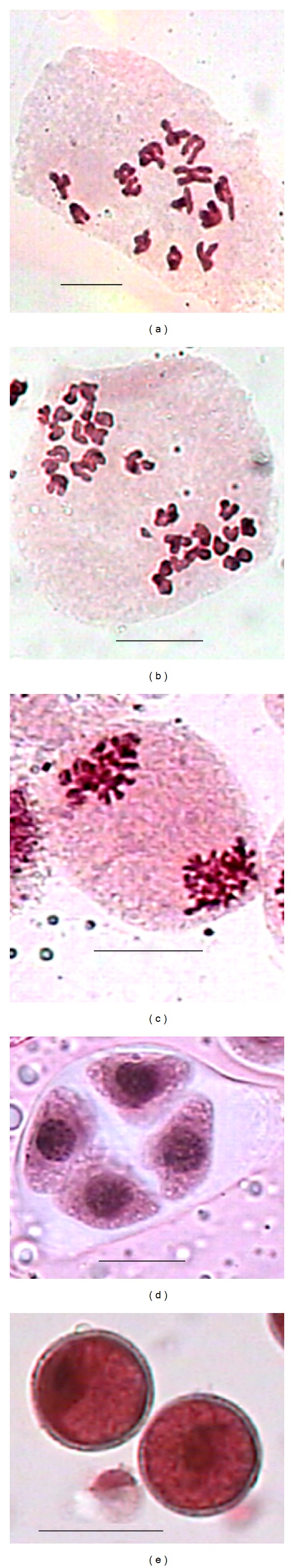
(a–e) Meiocytes with normal meiotic behaviour in *R. laetus*. (a) A PMC with 14 bivalents at diakinesis. (b) A PMC showing 14 : 14 chromosomes distributions at A-I. (c) A PMC showing two poles at A-I. (d) A tetrad with four *n* (reduced) microspores. (e) Apparently stained fertile *n* (reduced) pollen grains. Scale bar = 10 *μ*m (a–d); 20 *μ*m (e).

**Figure 3:**
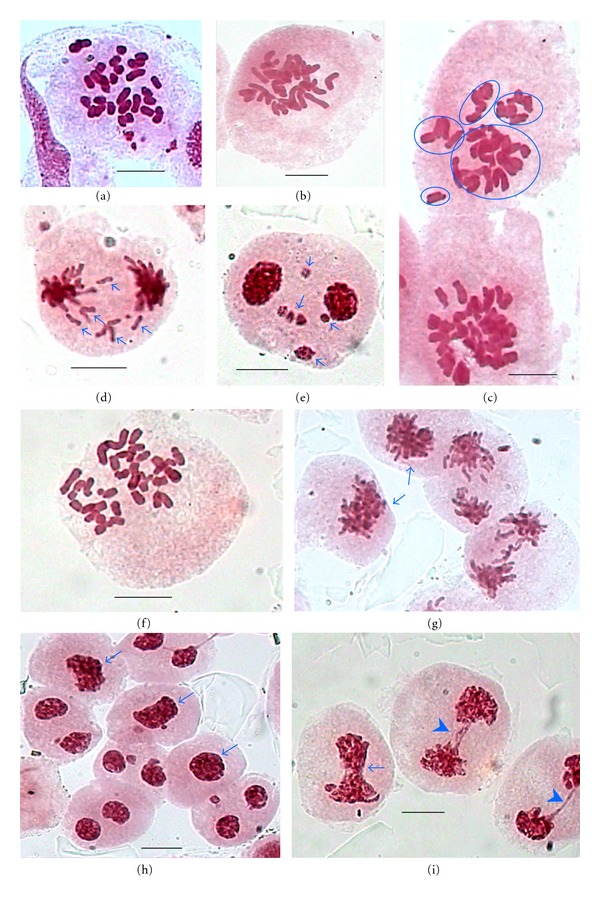
(a–i) Meiocytes with erratic male meiosis at first meiotic division in *R. laetus.* (a) A PMC with 28 randomly dispersed univalent chromosomes at M-I. (b) 28 univalent chromosomes positioned towards the periphery of PMC at M-I. (c) In one of the PMC univalent chromosomes lying in groups (encircled) and in an adjacent PMC in a single group in the centre. (d) A PMC at A-I with lagging chromosomes (arrowed). (e) Micronuclei at T-I (arrowed). (f) Unequal distribution of chromosomes (11 : 17) at A-I. (g) Restitution nuclei at A-I (arrowed). (h) Restitution nuclei at T-I (arrowed). (i) Thick (arrowed) and thin (arrowhead) chromatin bridge at T-I. Scale bar = 10 *μ*m.

**Figure 4:**
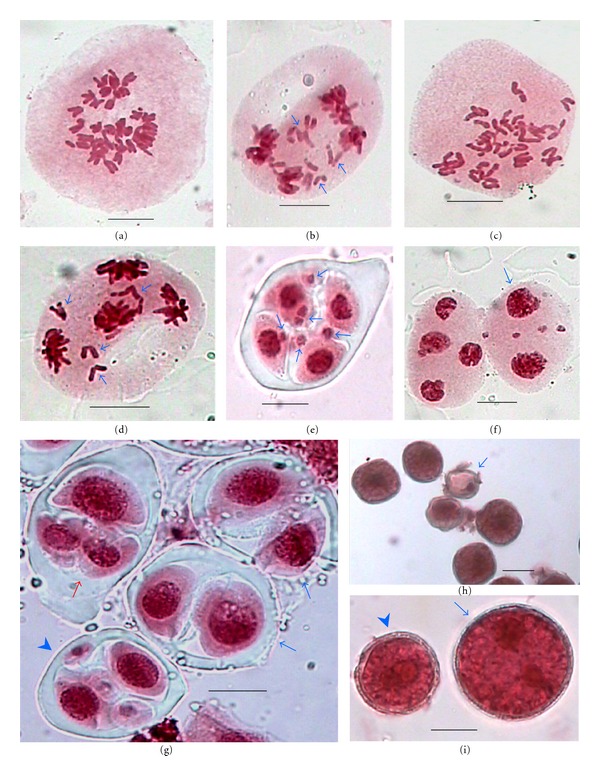
(a–i) Meiocytes with erratic male meiosis at second meiotic division in *R. laetus.* (a) Unequal distribution of chromosomes (10 : 18) at M-II. (b) Scattered chromosomes at anaphase-II (arrowed). (c) Random and unoriented distribution of chromosomes at M-II. (d) A PMC showing lagging of chromosomes (arrowed). (e) Tetrad with micronuclei (arrowed). (f) Out of the two PMCs, one showing restitution (unreduced) nuclei (arrowed) and the other with four haploid nuclei at T-II. (g) Sporads in group; dyads with micronuclei (arrowhead), dyads without micronuclei (blue arrows), and triads (red arrow). (h) Apparently stained fertile and unstained or lightly stained, and shriveled sterile (arrowed) pollen grains. (i) Apparently stained fertile *n* (reduced, arrowhead) and 2*n* (unreduced, arrowed) pollen grains. Scale bar = 10 *μ*m (a–d); 20 *μ*m (h).
